# Impact of Male Obesity on Semen Quality and Serum Sex Hormones

**DOI:** 10.1155/2012/407601

**Published:** 2011-11-30

**Authors:** Mamdoh Eskandar, Manal Al-Asmari, Suresh Babu Chaduvula, Mesfer Al-Shahrani, Mohammed Al-Sunaidi, Mona Almushait, Osman Donia, Suliman Al-Fifi

**Affiliations:** ^1^Department of Obstetrics and Gynecology and Reproductive Medicine, College of Medicine, King Khalid University, P.O. Box 641, Abha 61321, Saudi Arabia; ^2^Department of Obstetrics and Gynecology, Abha General Hospital, Abha, Saudi Arabia; ^3^Department of Pediatrics Endocrinology, College of Medicine, King Khalid University, Abha 61321, Saudi Arabia

## Abstract

* Introduction.* To investigate the association of high Body Mass Index (BMI) with semen parameters and reproductive hormones in men of reproductive age. *Setting.* The Saudi Center for Assisted Reproduction. *Method.* This study was conducted during the period from February 2009 to February 2011. Subjects were exposed through medical history evaluation as well as physical examination. BMI was calculated. Two semen samples about 1 week apart were taken from each participant by masturbation after 2–5 days of abstinence. The samples were assessed according to the WHO Criteria. Blood samples (5 ml) were withdrawn; centrifuged and the resulting sera were preserved at −4 degrees Centigrade. Serum FSH, LH, PRL, and Testosterone levels were estimated by the ELISA method. *Results.* There was no significant correlation between BMI and any of semen and hormonal parameters. There was significant negative correlation between age and total motility. Only the advanced paternal age has shown significant association with low motility (*P* = 0.007). *Conclusion.* Our study showed a significant effect of aging on sperm motility and concentration.

## 1. Introduction

Obesity is considered now as an epidemic disease that is rapidly progressing in developed and underdeveloped world. The effects of obesity not only relate to chronic medical conditions but also have been strongly related to reproductive problems [1, 2]. Potential effects of increased body mass index (BMI) in men on male fertility have not been subjected to the same degree of research as female obesity. There is growing evidence over the years that suggests a trend towards deterioration in semen quality in relation to obesity [3]. Several hypotheses have been proposed, and male obesity was suggested as a strong factor. Several studies have linked male obesity to poor semen quality and male infertility [4, 5]. The mechanisms that explain the relation between obesity and male infertility are not fully understood. Higher DNA fragmentation indexes in obese males [6], increased oxidative stress [7], and hormonal imbalance [8] have been suggested as possible mechanisms of obesity-associated subfertility. The aim of this study is to evaluate the effect of male obesity on semen quality and hormonal milieu. 

## 2. Materials and Methods

This is a retrospective study conducted at the Saudi Center for Assisted Reproduction, Abha, Saudi Arabia during the period from February 2009 to February 2011. A total of 500 males were recruited, those who were seeking fertility treatment in our IVF center. Local institute approval was taken before starting the study. Informed consents were taken from participating patients.

 Subjects were exposed to medical history evaluation as well as physical and systemic examination. Exclusion criteria included apparent genital infection, uncontrolled diabetes mellitus, uncontrolled hypertension, severe cerebrovascular or cardiovascular disease, and alcohol or drug abuse. BMI was calculated as the weight in kilograms divided by the square of height in meters. Height and weight are measured using the same scale for all participants.

Two semen samples about a week apart were taken from each participant by masturbation after 2–5 days of abstinence. The samples were assessed by 2 experienced personnel according to the WHO criteria [9].Venous blood samples (5 mL) were withdrawn from each subject and centrifuged and the resulting sera were preserved at −4 degree centigrade. Serum FSH, LH, PRL, and testosterone levels were estimated by the ELISA method (Diagnostics Systems Laboratories, Webster, Tex, USA).

Data were expressed as mean standard deviation (SD), median, percentage, and range. Comparison between groups was made by Mann-Whitney test. Correlation between different variables was made using Spearman‘s rank correlation. Multiple regression was used to analyze the relation between different values. *P* < 0.05 was considered as significant. MedCalc program was used for analysis.

## 3. Results

Five hundred males participated in the study. The characteristics of subjects are shown in Table 1. When subjects were classified into 2 groups based on BMI of <30 or >30, there was no significant difference between the 2 groups in any of the semen or hormonal parameters. These data are shown in Table 2.

There was no significant correlation between BMI and any of semen and hormonal parameters. There was significant negative correlation between age and total motility (CC = −0.138) and with a significant *P* value of 0.002. Table 3 shows the correlation among different parameters which were insignificant.

Several clinical and hormonal variables were entered in multiple regression analysis to detect relation to semen concentration. Only the age and sperm concentration showed significant association with a *P* value of 0.007. This data is shown in Table 4.

## 4. Discussion and Conclusions

The present study did not reveal any relation between BMI and any of semen or hormonal parameters of the studied population. This contradicts several studies which documented a deleterious effect of obesity on semen quality both in normal fertile [10, 11] and subfertile males [12, 13]. However, there are several recent reports that support our findings. In one meta-analysis, no relation between BMI and semen parameters was found [14]. Another study of healthy obese partners of subfertile couples failed to identify any relation between BMI and semen parameters [15]. Others suggested no effect of birth weight, childhood BMI, or adult BMI on semen quality [16]. It appears that this point is far from being settled. It needs larger studies with adequate control of confounders to reach a solid conclusion. Our study showed a significant effect of aging on sperm motility and concentration. There was a strong significant negative correlation of age and total motility percentage. Moreover, age was significantly associated with sperm concentration in the multiple regression model. These findings are supported with other studies which suggest deleterious effect of age on semen quality. One study documented a significant effect of age on all semen parameters [17]. It has also been suggested that the age of the patient is intimately related to a decrease of the number of sperms, with the decrease of the number of motile forms and the increase of the nonmotile [18]. This study adds to the opinion denying an effect of male obesity on semen quality or hormonal milieu.

## Figures and Tables

**Table 1:** Clinical hormonal and semen characteristics of subjects.

	Mean	SD	Median	Range
Age (Years)	34.77	7.67	33	21–68
BMI	28.12	6.10	27.70	16–50
FSH (mIU/mL)	7.06	6.02	4.9	0.57–28.90
LH (mIU/mL)	5.70	3.31	4.4	0.90–12.30
Testosterone (mmol/L)	6.44	4.15	4.20	2.20–28.90
PRL (ng/mL)	14.59	7.75	13	2.90–35.40
Total sperm count (millions/ejaculate)	150.96	178.87	87.70	0.00–980
Total sperm motility (%)	44.44	24.48	50	0.00–90
Sperm concentration (millions/mL)	59.52	63.78	40	0.00–380

**Table 2:** Clinical hormonal and semen parameters of the 2 groups.

	Nonobese males (BMI < 30)	Obese males (BMI ≥ 30)	*P* value
Number	322	178	
	Median (range)	Median (range)	
Age (years)	33 (21–68)	33 (63-22)	0.65
Concentration (millions/mL)	40 (0–300)	40 (0–380)	0.56
Semen volume (mL)	2.5 (0.20–7.70)	2.5 (0.20–8.20)	0.26
Total sperm motility (%)	50 (0–90)	49 (0–90)	0.90
Total sperm count (TSC) (millions/ejaculate)	81.5 (0–980)	93 (0–875)	0.29
Normal morphology (%)	17 (0–65)	17 (0–65)	0.63
FSH (mIU/mL)	4.5 (0.57–28.90)	5.6 (1.8-29)	0.22
LH (mIU/mL)	3.9 (0.90–12.3)	4.7 (0.90–12.3)	0.79
PRL (ng/mL)	13 (2.9–35.4)	12 (2.9–31.40)	0.26
Testosterone (mmol/L)	4.5 (2.20–28.90)	4.2 (2.90–28.90)	0.85

**Table 3:** Correlation of different variables.

		Age	BMI	Concentration	FSH	PRL	Testosterone	Total motility
Age	CC		0.005	0.078	0.019	0.033	−0.013	−0.138
	P		0.909	0.082	0.670	0.459	0.767	0.002*

BMI	CC			0.015	0.056	−0.082	−0.023	−0.013
	P			0742	0.214	0.067	0.601	0.774

Concentration	CC				0.039	0.012	−0.024	0.605
	P				0.380	0.786	0.589	0.08

FSH	CC					0.113	−0.114	−0.002
	P					0.11	0.11	0.960

PRL	CC						0.023	−0.020
	P						0.949	0.660

Testosterone	CC							−0.023
	p							0.614

CC: correlation coefficient; *significant *P* value.

**Table 4:** Multiple regression to predict concentration.

Independent variables	Coefficient	Srd. Error	*t*	*P* value
(Constant)	11.9576			
Age	1.0138	0.3778	2.684	0.0075*
BMI	0.01678	0.4678	0.0359	0.9714
LH	0.01528	0.3433	0.0265	0.8456
FSH	0.2616	0.4780	0.568	0.5701
Testosterone	−0.4797	0.6939	−0.691	0.4897
Prolactin	−0.2376	0.3698	−0.643	0.5207
